# Dynamic Modulus of Porous Asphalt and the Effect of Moisture Conditioning

**DOI:** 10.3390/ma12081230

**Published:** 2019-04-15

**Authors:** Tan Hung Nguyen, Jaehun Ahn, Jaejun Lee, Jin-Hwan Kim

**Affiliations:** 1 Department of Civil and Environmental Engineering, Pusan National University, Busan 46241, Korea; nthung010189@gmail.com; 2 Department of Civil Engineering, Chonbuk National University, Jeonju-si 54896, Korea; lee2012@jbnu.ac.kr; 3 Korea Expressway Corporation Research Institute, Hwaseong-si 18489, Korea; kimtopia@ex.co.kr

**Keywords:** dynamic modulus, porous asphalt, moisture susceptibility, ratio of modulus, freeze-thaw cycle

## Abstract

Porous asphalt has been used for permeable pavement to improve safety of roadways and the effectiveness of storm water management. As a surface drainage layer with frequent exposure to water, this material is affected by moisture. In this study, dynamic modulus tests were performed on both moisture unconditioned and conditioned specimens to characterize viscoelastic properties of porous asphalt mixture. The dynamic modulus values of porous asphalt materials with air void content of 9.0% and 20.5% were investigated at dry condition and after specified moisture conditioning cycles. One cycle of moisture conditioning procedure included placing specimens in water tank at 60 °C for 24 h, and then in another water tank at 25 °C for additional 2 h. The results showed that porous asphalt mixture with lower air void content resulted in higher values of dynamic modulus, and these values of porous asphalt with air void content of 9.0% was about 1.5 to 3.0 times that of porous asphalt with air void content of 20.5%. Higher value of the first number of performance graded binder (average 7-day maximum pavement design temperature) seems to make the dynamic modulus values at high temperatures larger. After moisture conditioning, the dynamic modulus of porous asphalt mixture increased, overall, especially at low temperatures. The appropriated selection of asphalt binder, a weakening of asphalt due to moisture damage can be reduced.

## 1. Introduction

Dynamic modulus (DM) measures the stiffness and viscoelasticity of an asphalt concrete material, and is used for the structural design of flexible pavements. The master curve of DM is a critical input in the mechanistic empirical design. The DM of asphalt mixture is sensitive to moisture, and therefore may be used to evaluate the deterioration of asphalt due to moisture as attempted by other researchers including the National Cooperative Highway Research Program (NCHRP) project 9-34 [1]. The benefits of investigating the effect of moisture by using DM are as follows: (1) the effect of moisture on DM can directly be implemented in the design as DM itself is a design input [1]; (2) as the DM test is non-destructive, a single sample with and without moisture damage can be measured, thus avoiding the influence of sample-to-sample variation on the results; and (3) DM is the result of dynamic loading which simulates actual traffic loading conditions better than static loading [2].

Research has been carried out to evaluate the effect of moisture conditioning on the viscoelastic properties of asphalt mixtures based on DM test. Zhang et al. [3] observed the behavior of two typical porous asphalt (PA) mixtures were designed by the Virginia Department of Transportation (VDOT) and the Oklahoma Department of Transportation (OKDOT). Specimens with different air void content (%AV), 20%, 25%, and 28% for VDOT mixture and 20%, 23%, and 25% for OKDOT ones, were fabricated. When they compared DM of specimen with and without moisture conditioning at 25 °C, the reduction in DM tended to be larger as the %AV increased. In cold regions, where asphalt pavement was significantly affected by a wide range of seasonal temperature variations, the evaluation of deterioration was carefully considered in the design. El-hakim and Tighe [4] tested three conventional mixtures and a modified one (with asphalt content 0.8% higher than the optimum values). Specimens were evaluated at dry and after being subjected to freeze-thaw moisture conditioning. The results showed that DM displayed a noticeable decrease at low temperatures for the conventional mixtures, but not for the modified mixture. These findings indicated that the additional asphalt content (%AC) can result in less deterioration of asphalt material due to moisture. Lee et al. [5] investigated the behavior of dense-graded hot mix asphalt (HMA), stone matrix asphalt (SMA) and PA mixtures under thawing conditions. It was found that after moisture conditioning, the DM decreased significantly for the HMA mixtures, whereas it slightly increased for the SMA and PA mixtures. They concluded that with the asphalt binder (PG82-22), the PA mixture increased its resistance to moisture.

The percent retained of stiffness after moisture conditioning, referred to as DM ratio (DMR) [2] has been used as an alternative index to the tensile strength ratio (TSR). Nadkarni et al. [2] studied the resistance to moisture damage of dense-graded, gap-graded, and open-graded mixtures. The results revealed that DMR of all mixtures decreased after freeze-thaw moisture conditioning, the open-graded mixtures showed the largest decay in DM. They, however, explain low DMR values of open-graded specimens may be partially attributed to harder and poorer handling of specimens with high %AV during moisture conditioning. Badeli et al. [6] investigated DMR values of asphalt mixtures at different moisture conditioning cycles. They mixed the specimens with %AV of 2%, 3.5%, and 7%, and measured DMR for those after undergoing through only saturation, or 150 or 300 cycles of freeze-thaw. Based on the results of the analysis, it was concluded that the stiffness of all mixtures increased after saturation at high temperatures. After 150 freeze–thaw cycles, DMR of all mixtures decreased at high temperatures (low loading frequencies). Finally, after 300 freeze–thaw cycles, all mixtures displayed a noticeable decrease in DMR because of the stripping issue. They concluded that the deterioration due to freeze–thaw moisture conditioning increased as %AV of the mixture increased.

Generally, moisture conditioning considerably affects the performance of asphalt materials and is one of the major sources of pavement distresses. DM tests were conducted with unconditioned and conditioned moisture specimens to provide a potential solution to evaluate the viscoelastic properties of asphalt material due to moisture damage. PA material is often used as a surface layer in permeable pavement that stores storm water in the pore space, or a friction course over dense-graded asphalt layer in a highway application which only allows water to be drained in the pore. Because PA material is constantly exposed to moisture, it is considered to have a risk of moisture-induced distress. More attention should be paid to figure out the resistance of different PA materials to moisture damage. The objective of this study is to investigate the viscoelastic properties of PA materials and the effect of moisture conditioning. DM tests were conducted with specimens at dry condition and after specified moisture conditioning cycles, DMR values were investigated.

## 2. Experimental Program

### 2.1. PA Specimens

The PA mixture is often made with open-graded aggregates, so that the material has a higher content of interconnected air void than traditional ones. In this study, PA specimens with %AV (or porosities) of 9.0% and 20.5%, symbolized by PA10 and PA20, respectively, were manufactured and investigated. The specimens were designed and made based on Korean Standard (KS) F 2349 “Hot mix asphalt mixture” [7]. An asphalt binder, PG82-34, widely used for PA in South Korea, was used. The properties of asphalt binder are presented in Table 1.

**Figure 1:**
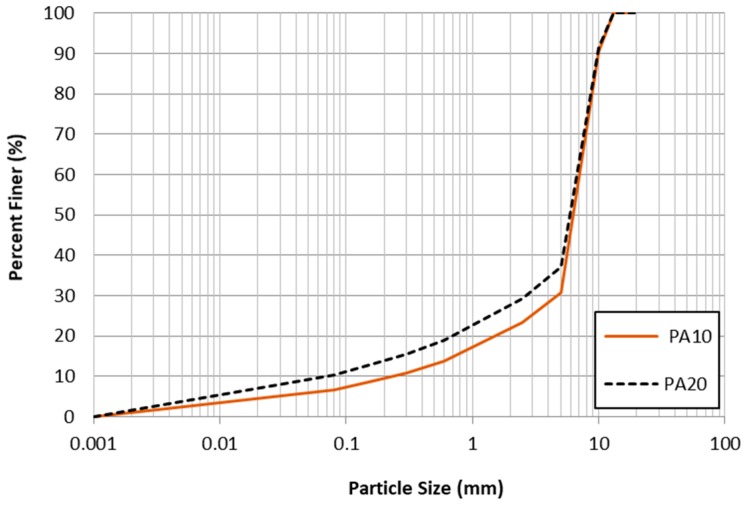
Aggregate gradations of PA10 and PA20.

Aggregates for both PA10 and PA20 specimens were collected from the same quarry and the gradations of the two mixtures were determined based on the requirements for PA in Korea. The nominal maximum aggregate size (NMAS) of the two mixtures was designed to meet the specifications of 10 mm. Although the mixture designs of specimens are very similar, they were designed independently. The aggregate gradations of PA10 and PA20 specimens are presented in Figure 1.

Draindown and Cantabro Loss tests were performed for mixtures according to KS F 2489 [9] and KS F 2492 [10]. Maximum theoretical specific gravity (G_mm_) values of PA10 and PA20 specimens were determined to be 2.434 and 2.445, respectively. Summary of properties of PA mixtures are displayed in Table 2. It is noted that in KS F 2492 [10], the Cantabro Loss is measured at both −20 °C and 20 °C.

### 2.2. Equipment

**Figure 2:**
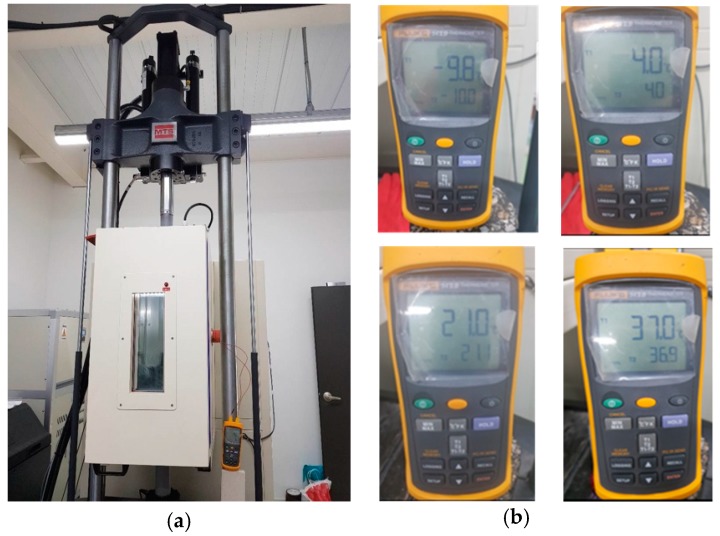
Equipment: (**a**) MTS system with temperature chamber; (**b**) thermometer.

The tests were performed with a Material Testing Systems (MTS) 370.10 servo-hydraulic testing system, in Figure 2a, which includes a load frame rated at 100-kN with 150-mm stroke actuator and 25-kN load cell [11]. The actuator is capable of inducing cyclical axial loads. In this study, a temperature chamber was assembled to the MTS unit and used to apply the desired temperature to the specimens. The heating rate of the chamber was 1.5 °C/min, and temperature application with a range from −50 °C to 100 °C. The DM test system included linear variable differential transformers (LVDT) stud placer unit, upper and bottom platens with 100-mm diameter, and two LVDTs with 100-mm length manufactured by Cooper Technology Company (Ripley, United Kingdom) [12]. In addition, a thermometer, in Figure 2b, was used to measure the temperature of dummy specimen before and during the experiment. Two duplicate specimens for each mixture were prepared and stored as suggested by the section for specimen preparation in AASHTO TP342 [13].

### 2.3. Test Setup

The specimen was set up in the temperature chamber between two platens. To reduce the friction between platens and specimen, two Teflon papers were placed. The DM test was conducted at temperatures of −10 °C, 4 °C, 21 °C, 37 °C, and 54 °C and frequencies of 25 Hz, 10 Hz, 5 Hz, 1 Hz, 0.5 Hz, and 0.1 Hz. However, at 54 °C, the LVDT supporters were unstable, and the results could not be interpreted. As such, the results at 54 °C were not used in the analysis. For each testing temperature level, specimens were conditioned in the temperature chamber for the required time to reach equilibrium based on AASHTO TP342 [13]. To verify the testing temperature, the temperature of a dummy specimen that had the same size with the test specimen was measured by an independent thermometer. The temperature tolerance was controlled for the experiment to be ±0.3 °C at each temperature level. The magnitude of uniaxial sinusoidal stress was chosen to maintain the micro-strain from 50 to 150. The DM test was started from the lowest temperature and proceeded to the highest temperature (from −10 °C to 37 °C). At a specific temperature, the loading frequencies were applied from the highest to the lowest (25 Hz to 0.1 Hz). Vertical deformations were obtained using two LVDTs with 100 mm in length which were mounted to the specimen. The data acquisition program was set to collect at least 50 readings of load and deformations per cycle.

**Figure 3:**
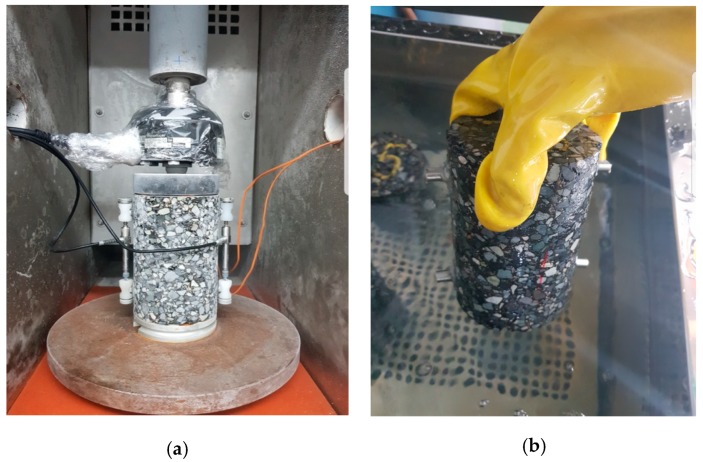
Specimen setup: (**a**) DM test; (**b**) moisture conditioning.

DM tests were conducted on the same specimens before moisture conditioning, after the first cycle of moisture conditioning, and the after the second cycle. The idea behind this study was to follow the conditioning approach in AASHTO T283 [14] but without freezing step. One cycle of moisture conditioning procedure included thawing in water and drying. For thawing, all specimens were placed in water tank at 60 °C for 24 h, and then in another water tank at 25 °C for additional 2 h. To minimize the effect of water pressure during conditioning on the specimens, they were put under a constant level of water. Finally, specimens were taken and dried in a temperature chamber at 25 °C for 16 h. The DM tests were immediately conducted after drying (Figure 3).

## 3. Dynamic Modulus and the Effect of Moisture Conditioning

### 3.1. Dynamic Modulus

The DM master curve was obtained based on Equations (1) and (2) at a reference temperature of 21 °C following NCHRP 1-37A [15]:

(1) $$\log\left|E^{*}\right|=\delta +\frac{\alpha }{1+e^{\beta +\gamma (\log\omega_{r})}}$$

(2) $$\omega =\frac{\omega_{r}}{a(T)}$$

where E* is dynamic modulus; *δ* is minimum value of E*; *δ* + *α* is maximum value of E*; *β*, *γ* are parameters describing the shape of the sigmoidal function; *ω_r_* is reduced frequency of loading at the reference temperature; *ω* is frequency of loading at a given temperature; *α*(*T*) is shift factor as a function of temperature; and *T* is temperature.

**Figure 4:**
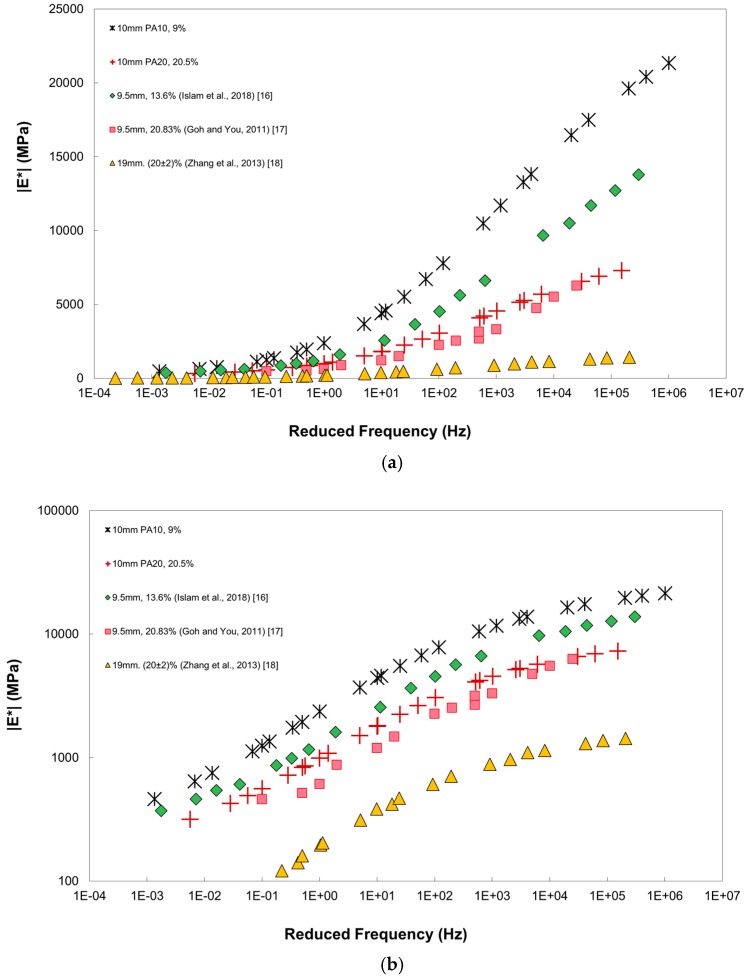
Dynamic Modulus master curves of unconditioned specimens in: (**a**) semi-log scale; (**b**) log-log scale.

**Figure 5:**
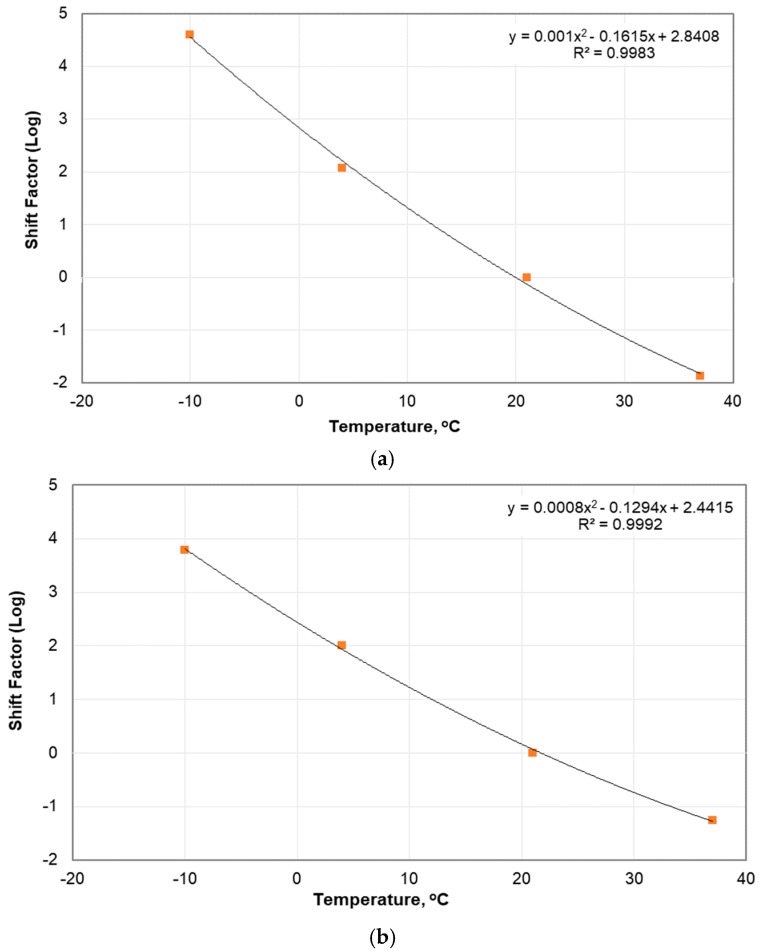
Shift factors for: (**a**) PA10; and (**b**) PA20.

The DM master curves for the unconditioned specimens are displayed in Figure 4a,b with master curves of other PA mixtures from Islam et al. [16], Goh and You [17], and Zhang et al. [18] whose material properties are described in Table 3. The shift factors used to generate the master curves are presented in Figure 5. The results of PA10 and PA20 compare well with the results from literatures. It is noted that the PA mixture with lower %AV results in higher values, which has also been reported by Zhang et al. [3]. When the DM values of PA10 and PA20 were compared, the DM of PA10 was about 1.5 to 3.0 times that of PA20, with larger difference at lower temperature. The DM master curves of PA20 and Goh and You [17] compared well, since their %AV, NMAS, and %AC are similar. The first number of PG (average 7-day maximum pavement design temperature) of PA20 was higher than that of Goh and You [17], and therefore PA20 showed larger DM values at high temperatures (low loading frequencies). When the results of PA20, Goh and You [17] and Zhang et al. [18] were compared, the DM from Zhang et al. [18] presented significantly smaller values, even they had similar %AV (20%). In addition, PA20 for this study with NMAS of nearly 10 mm displayed higher DM values than those from Zhang et al. [18] with NMAS of 19 mm, although they were designed with same %AV. At a glance, the DM values of specimen with large NMAS seem to be lower; however, the effect of NMAS on DM did not result the same tendency as reported [5,19]. The effect of NMAS on DM of PA materials should be investigated further.

### 3.2. Effect of Moisture Conditioning

**Figure 6:**
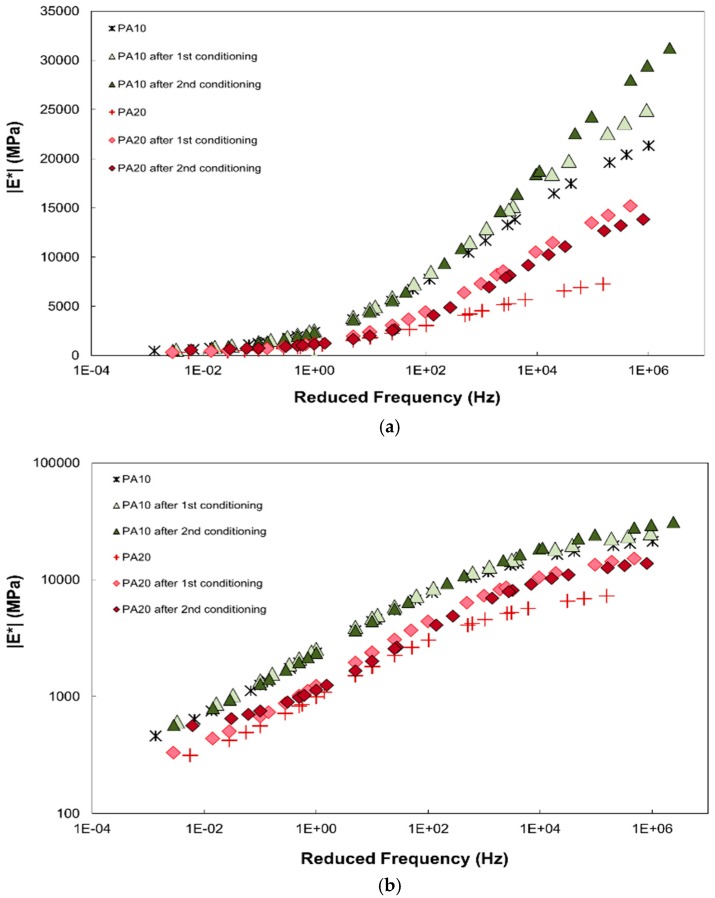
Dynamic Modulus master curves of unconditioned and conditioned specimens in: (**a**) semi-log scale; (**b**) log-log scale.

The DM master curves of moisture unconditioned and conditioned specimens are presented in Figure 6. After moisture conditioning, the DM of PA mixture increased, overall, especially at low temperatures. In fact, the rise in stiffness values after moisture conditioning has been reported in literatures [3,5,6,20,21]. Coplantz and Newcomb [20] addressed that without freeze-thaw cycling, the deterioration may not be enough to reduce the performance of asphalt mixtures. Lottman [21] reported that the increase in stiffness of asphalt may happen at the early age of water-saturated asphalt specimen. The increase in DM may be attributed to pore water captured in closed void [5,6]; the excess pore water pressure may be generated under test loading. Bausano and Williams [22] concluded that with the appropriated selection of asphalt binder, a weakening of asphalt due to moisture damage can be reduced. In this study, during the experiment, no coated aggregate was detached from the specimens after each moisture conditioning cycle. Further researches should be conducted to investigate the impact of moisture conditioning on the PA mixtures.

**Figure 7:**
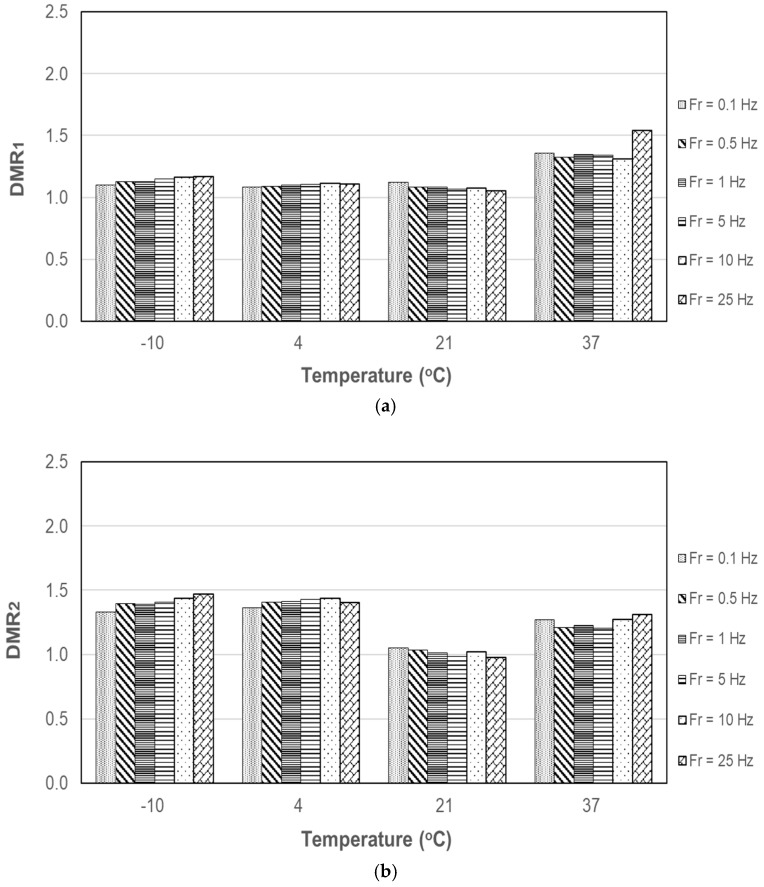
DMR of PA10: (**a**) after first cycle conditioning; (**b**) after second cycle conditioning.

**Figure 8:**
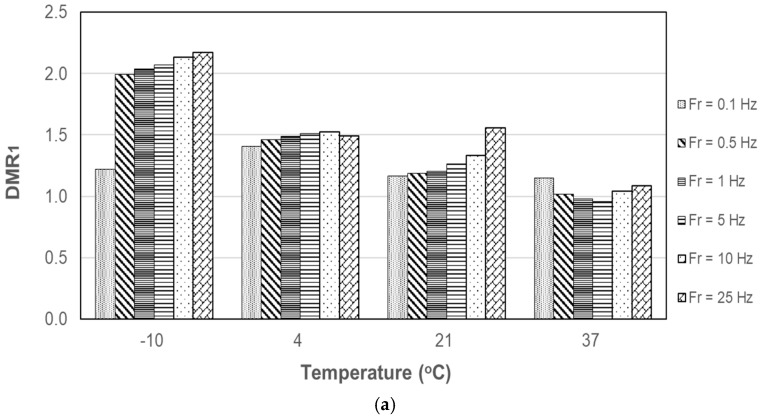

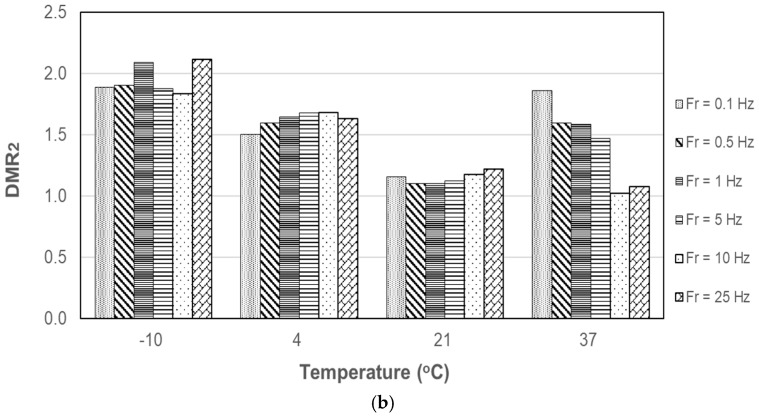
DMR of PA20: (**a**) after first cycle conditioning; (**b**) after second cycle conditioning.

The ratio of dynamic modulus (DMR) was analyzed by using Equation (3):

(3) $$\mathrm{DMR}=\frac{E_{\mathrm{condition}}}{E_{\mathrm{uncondition}}}$$

where E_condition_ and E_uncondition_ are the DM of moisture conditioned and unconditioned specimens, respectively. DMR of PA10 and PA20 are displayed in Figure 7 and Figure 8 with respect to temperature and loading frequency. DMR_1_ and DMR_2_ represent DMR after the first and second cycle of moisture conditioning, respectively. Overall, DMR of PA10 is not very dependent on the loading frequency as observed in NCHRP Project 9-34 [1]. PA20 shows more variability with respect to loading frequency. 

## 4. Conclusions

The DM values of PA materials with %AV of 9.0% and 20.5% were investigated at dry condition and after specified moisture conditioning cycles. The specimens were comprised of NMAS of 10 mm and PG82-34 binder. One cycle of moisture conditioning procedure included placing specimens in water tank at 60 °C for 24 h, and then in another water tank at 25 °C for additional 2 h.

PA mixtures with lower %AV resulted in higher values of DM, and the DM of PA with %AV of 9.0% was about 1.5 to 3.0 times that of PA with %AV of 20.5%. Higher value of the first number of PG (average 7-day maximum pavement design temperature) seems to make the DM values at high temperatures larger.

After moisture conditioning, the DM of PA mixtures increased, overall, especially at low temperatures. The increase in DM may be attributed to pore water captured in closed void. During the experiment, no coated aggregate was detached from the specimens after each moisture conditioning cycle. The appropriated selection of asphalt binder, a weakening of asphalt due to moisture damage can be reduced. DMR of PA with %AV of 9.0% was not dependent on the loading frequency; DMR of PA with %AV of 20.5% showed more variability with respect to loading frequency. 

The effect of NMAS and moisture on DM should be further investigated to incorporate these effect in design.

## Figures and Tables

**Table 1:** Properties of asphalt binder PG82-34 [8].

Test	Value	Unit
Viscosity (135 °C)	3.4	Pa·s
G*/sinδ (Original) (82 °C)	1.27	kPa
G*/sinδ (After RTFO) (82 °C)	2.49	kPa
G*·sinδ (After PAV) (28 °C)	564	kPa
Flash Point	342	°C
Stiffness (−24 °C)	194	MPa
m-value (−24 °C)	0.32	°C

**Table 2:** Properties of PA10 and PA20.

Mixture		PA10	PA20
Asphalt content (%)		6.1	5.9
Air void content (%)		9.0	20.5
Draindown (%)		0.15	0.19
Cantabro Loss (%)	−20 °C	12.29	43.9
	20 °C	0.64	10.91

**Table 3:** Properties of PA (after Goh and You, 2011, Zhang et al., 2013 and Islam et al., 2018 [16,17,18]).

Mixture	Goh and You, 2011	Zhang et al., 2013	Islam et al., 2018
Binder	58–34	76–22	70–28
NMAS (mm)	9.5	19	9.5
%AC	5.75	6	5.0
%AV	20.83	20 ± 2	13.6
